# Mental Health Outcomes and Digital Service Utilization: A Comparative Analysis of Arab American and Arab/Middle Eastern International Students During the COVID-19 Recovery Period

**DOI:** 10.3390/healthcare13192436

**Published:** 2025-09-25

**Authors:** Fatima M. Aldarweesh, Christopher E. Johnson, David J. Roelfs, Seyed M. Karimi, Demetra Antimisiaris

**Affiliations:** 1 Department of Health Management and Systems Sciences, School of Public Health and Information Sciences, University of Louisville, Louisville, KY 40202, USA; seyed.karimi@louisville.edu (S.M.K.); demetra.antimisiaris@louisville.edu (D.A.); 2 Institute of Health Administration, College of Business, Georgia State University, Atlanta, GA 30303, USA; cjohnson517@gsu.edu; 3 Department of Sociology, College of Arts and Sciences, University of Louisville, Louisville, KY 40292, USA; david.roelfs@louisville.edu

**Keywords:** Arab American, international students, loneliness, digital mental health, depression, anxiety, perceived need

## Abstract

**Background**: During COVID-19 Arab American and Arab/Middle Eastern (AME) international students faced disproportionately higher psychosocial stressors like other racial populations that may influence their mental health and help-seeking behaviors. Objective: This study examined and compared the prevalence of depression, anxiety, and loneliness among Arab American and AME international college students in addition to how loneliness is associated with perceived mental health need and the use of digital mental health (DMH) services. **Methods**: Data from the 2022–2024 Healthy Minds Study (HMS) comprised a final analytic sample of 3249 AME students (2662 Arab American; 587 AME international). Logistic regression and generalized structural equation modeling (GSEM) were used to examine associations and mediating pathways. **Results**: Depression and anxiety were prevalent among Arab American students (44.3% and 40.0%) compared to AME international students (40.9% and 37.0%). Rates of loneliness were similar (56.2% vs. 56.9%). Loneliness was bidirectionally associated with both depression and anxiety (*p* < 0.001). Perceived need for care emerged as a significant mediator linking psychological symptoms to DMH service use (OR = 2.56, *p* < 0.001), while loneliness did not directly predict DMH utilization. Only 10% of students reported using DMH services. **Conclusions**: Mental health disparities among AME students exist. Institutions should prioritize culturally responsive approaches to support the mental health needs of diverse student populations.

## 1. Introduction

The post-COVID-19 recovery period offers a critical window for assessing the ongoing psychological impacts of the pandemic on college students, particularly those from culturally marginalized and historically underserved groups. Among these students are Arab/Middle Eastern (AME) individuals, both domestic and international. These groups represent distinct cultural and structural communities shaped by specific sociocultural, institutional, and immigration-related experiences that may have influenced how they were affected by the pandemic’s mental health consequences.

During 2022–2023, more than 38,000 international students from the Middle East and North Africa (MENA) region were enrolled in colleges and universities in the United States (U.S.) [1]. This group made up nearly 4% of the entire international student population in the U.S. [2]. In addition, the U.S. is home to roughly 3.7 million Arab Americans, many of whom are of traditional college age, i.e., 18–24 years old. A large number of these students are enrolled in higher education, especially in states such as Michigan, California, New York, and Illinois [3]. Although their presence in higher education is significant, AME students continue to be underrepresented in mental health research and often encounter barriers to accessing adequate campus support services [4,5]. Prior studies have documented that they experience higher rates of anxiety and depression, partly due to racial discrimination, Islamophobia, and systemic marginalization within U.S. educational settings [6,7,8].

While Arab Americans and AME international students may share ethnic roots, their lived experiences within and beyond the U.S. education system can shape their mental health in very different ways. AME international students are at greater risk for depression, anxiety, and loneliness due to the stress of adapting to a new culture, limited access to support networks, and separation from their families and home communities [9,10].

Studies conducted during the COVID-19 pandemic highlighted loneliness as a significant concern for young adults. One national study of U.S. young adults during COVID-19 revealed high levels of loneliness, which were strongly linked to mental health struggles such as anxiety and depression and substance use [11]. While these issues were widespread among college students during the pandemic, the intensity of loneliness and its impact varied across different student groups [12,13,14]. Similar patterns have been reported among international students in other contexts; for example, a systematic review and meta-analysis of Chinese international students during COVID-19 documented elevated depression and anxiety, underscoring the global salience of these concerns [15]. Moreover, these mental health challenges have also been observed in other countries, including the UK and Australia, where international students report elevated rates of depression, anxiety, and loneliness, often linked to acculturative stress, social isolation, and barriers to accessing culturally appropriate care [16,17]. Despite this international evidence, very few studies have explicitly focused on Arab American or AME international students in this context, leaving a major gap in understanding how loneliness manifests in these culturally specific populations. Some researchers have described this situation as part of a broader “social recession,” calling attention to the widespread impact of social isolation on young people’s mental health. This highlights the need for more detailed research on how loneliness affects students from diverse backgrounds [18,19,20].

Although there are well-documented differences in mental health service use among racial and ethnic groups in college settings, there is limited data on how AME students seek out mental health care, especially through digital services. This gap is particularly important because many mental health services shifted to virtual formats during the pandemic. As digital tools become an essential part of campus mental health support, it is critical to understand who is using them and how they are accessed by different groups. Study aims. This study (i) compares the prevalence of depression, anxiety, and loneliness between Arab American and AME international students; (ii) tests bidirectional associations between loneliness and depression/anxiety; and (iii) evaluates whether perceived need mediates the association between symptoms and digital mental health (DMH) service use.

### Research Hypotheses

**H1.**  *Arab American and AME international students will differ in their prevalence of depression, anxiety, and loneliness.*

**H2.**  *Loneliness will show a bidirectional association with depression and anxiety.*

**H3.**  *Perceived need will mediate the relationship between mental health symptoms and DMH service use.*

## 2. Materials and Methods

### 2.1. Data Source and Sample Selection

This study used secondary data from the 2022–2024 cycle of the Healthy Minds Study (HMS), a national online survey of mental health, health behaviors, and service use among U.S. college and university students [21]. The HMS employs validated instruments, including the Patient Health Questionnaire-9 (PHQ-9) and the Generalized Anxiety Disorder 7-item scale (GAD-7). Core modules collect data on demographics, mental health conditions, and service utilization, while participating institutions may add optional modules tailored to their needs.

The HMS sample is large and diverse, covering approximately 124 campuses. At each institution, about 4000 students are randomly selected to participate [22]. With Institutional Review Board (IRB) approval, this study incorporates administrative data supplied by participating institutions. To ensure representativeness, non-response propensity weights were applied using institutional records (e.g., gender, race/ethnicity, academic level, and GPA), consistent with HMS protocols. Due to privacy and ethical considerations, HMS data are restricted but may be accessed upon request and with permission from the Healthy Minds Network [23]. IRB approval for the use of these secondary data was obtained from the University of Louisville (IRB Number: 25.0308; Approval Date: 5 August 2025).

**Figure 1:**
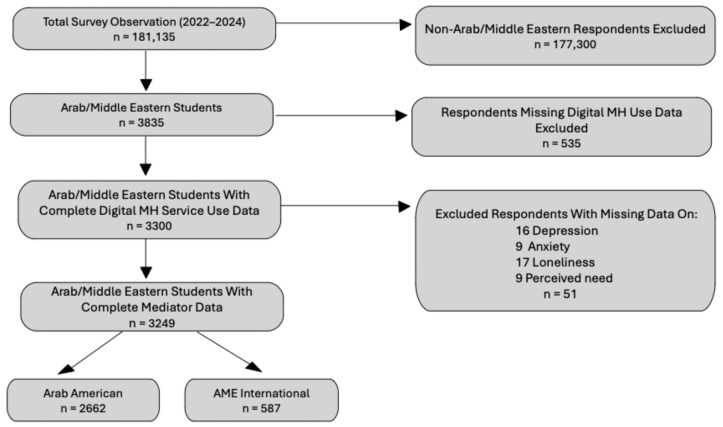
Study Missing Observations Flow Diagram.

Figure 1 demonstrates the sample selection process. A total of 181,135 survey responses were collected. Of these, 177,300 non-AME respondents were excluded, retaining a subsample of 3835 AME students. Among these, 535 were removed due to missing data on the key outcome variable, DMH service use. Of the remaining 3300 students, another 51 were removed due to incomplete responses on at least one of the following variables: depression (*n* = 16), anxiety (*n* = 9), loneliness (*n* = 17), and perceived need for mental health care (*n* = 9).

The final analytic sample included 3249 AME students with complete data on all study variables. Based on self-reported citizenship and visa status, this sample was further divided into two groups: Arab American (*n* = 2662) and AME international students (*n* = 587). Group-level comparisons were conducted only for Hypothesis 1, whereas Hypotheses 2 and 3 were tested across the full Arab/Middle Eastern student sample (combining both groups).

### 2.2. Conceptual Framework

This study’s conceptual framework was guided by Andersen’s Behavioral Model of Health Services Use, which is a widely used theoretical framework for examining the factors influencing health care utilization [24]. The model identifies three primary domains that shape service use: predisposing factors (e.g., demographic characteristics), enabling factors (e.g., awareness of services or insurance coverage), and need-based factors (e.g., perceived or evaluated illness severity). In this study’s adapted framework, mental health indicators, including depression, anxiety, and loneliness, were conceptualized as need-related factors, which influence students’ perceived need for care and ultimately their utilization of DMH services. Perceived need, defined as a student’s self-acknowledged need for emotional or mental health support, was modeled as a proximal mediator between psychological symptoms and engagement with DMH services [25]. Enabling factors, including perceived social support, awareness of campus services, and insurance coverage, function as a structural element that can either promote or hinder access to mental health resources [26,27]. Predisposing factors, such as age, gender, degree level, race, and international student status, reflect background characteristics that may influence students’ mental health beliefs, prior experiences with care, and cultural perceptions of help-seeking [26,27,28].

**Figure 2:**
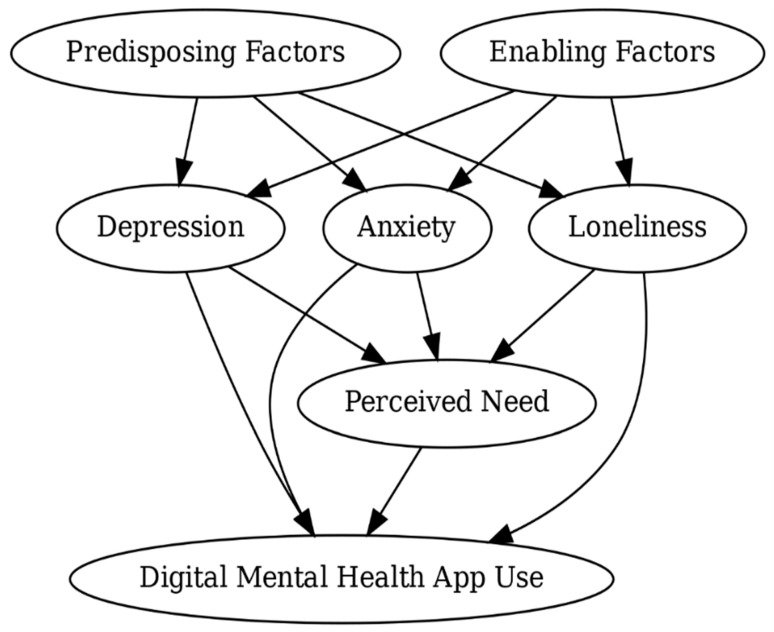
Adapted Conceptual Framework Based on Andersen’s Model Illustrating the Role of Loneliness, Perceived Need, and Digital Mental Health Service Utilization.

Furthermore, loneliness is viewed not just as a symptom of mental health need but also as a factor that can shape how students respond to that need. It may influence whether, and how strongly, perceived need leads to seeking help. This approach is informed by empirical research showing the bidirectional relationship between loneliness and mental health conditions, such as depression and anxiety [29]. This conceptual approach builds on prior applications of Andersen’s model in studies focused on digital health and college student populations [24,25,26]. Figure 2 illustrates the proposed conceptual relationships among these variables.

### 2.3. Measures

#### 2.3.1. Digital Mental Health Services Utilization

Digital mental health (DMH) service utilization was assessed using up to three items available across survey datasets: (1) “Have you ever used a smartphone app to manage your wellness or mental/emotional health?” (2) “Do you utilize any self-guided therapy resources through a phone app or website?” and (3) “Have you ever used a mobile app that provides resources or services for mental health support (e.g., BetterHelp, Calm, Headspace, TalkCampus)?” Because not all datasets included all three items, a standardized binary indicator was created to reflect DMH use across all datasets. The coding followed a hierarchical logic: when the first item was available, responses of “Yes” were coded as 1 (indicating use), and “No” as 0. If this item was unavailable or missing, responses to the second item were used, with “Yes” coded as 1 and “No” as 0. If neither of the above items indicated use, the third item was considered, with “Yes” coded as 1 and “No” coded as 0. Responses of “Prefer not to say” were treated as missing. This stepwise process enabled the integration of DMH utilization indicators across multiple survey waves and instruments. Missing values in the constructed variable were retained during initial processing, and cases with missing DMH use were excluded from the final analysis to ensure data completeness for multivariable modeling.

#### 2.3.2. Depression (PHQ-9)

Depression status was assessed using the PHQ-9, a widely validated nine-item questionnaire that asks the following: “Over the last two weeks, how often have you been bothered by any of the following problems?” The items cover symptoms such as lack of interest or pleasure, feeling down or hopeless, sleep issues, fatigue, appetite changes, low self-worth, difficulty concentrating, psychomotor changes, and suicidal thoughts. Each item was answered on a four-point scale: 1 = Not at all, 2 = Several days, 3 = More than half the days, and 4 = Nearly every day. To prepare the data for analysis, responses were recoded from 1–4 into numerical scores ranging from 0–3, aligning with standard PHQ-9 scoring practices. A composite variable, phq9_total, was created by summing the recoded scores across all nine items. A cutoff score of 10 or higher on the PHQ-9 was used to indicate moderate to severe depressive symptoms, consistent with established clinical and research guidelines [30]. A binary variable, depression_status, was then created: students with a total score of 10 or more were coded as 1, while those scoring below 10 were coded as 0.

#### 2.3.3. Anxiety (GAD-7)

Anxiety status was measured using the GAD-7, a validated seven-item screening tool for generalized anxiety disorder. Participants were asked the following: “Over the last two weeks, how often have you been bothered by the following problems?” The items addressed symptoms such as nervousness, excessive worrying, restlessness, irritability, and fear of impending doom. Response options were as follows: 1 = Not at all, 2 = Several days, 3 = Over half the days, and 4 = Nearly every day. Each item was recoded into a numerical scale from 0 to 3, in line with standard GAD-7 scoring. A total score, gad7_total, was calculated by summing the values across all seven items. A score of 10 or higher indicated moderate to severe anxiety, based on widely accepted research thresholds [31]. A binary variable, anxiety_status, was then created: respondents scoring 10 or above were coded as 1, while those scoring below 10 were coded as 0. This binary variable allowed for the identification of students at risk for clinically significant anxiety symptoms.

Consistent with established psychometric guidelines and Healthy Minds Study protocols, participants were included in PHQ-9 analyses if they answered at least 7 of the 9 items [23]. When 1 or 2 items were missing, those missing values were imputed using the mean of the completed items to preserve scoring validity. For the GAD-7, a minimum of 6 completed items was required, with similar mean imputation applied for up to 1 missing item. Respondents who did not meet these minimum criteria were excluded from depression- or anxiety-related analyses to ensure data integrity and adherence to scoring standards [23,32].

#### 2.3.4. Loneliness (UCLA 3-Item Scale)

Loneliness was measured using the UCLA 3-item Loneliness Scale, a validated instrument assessing subjective feelings of social isolation and lack of companionship. Participants responded to three questions: how often they feel (1) lacking companionship, (2) left out, and (3) isolated from others, using a 3-point scale (1 = “Hardly ever,” 2 = “Some of the time,” 3 = “Often”). A composite loneliness score (lonesc) was computed by summing the three items (range: 3–9), with higher scores indicating greater loneliness. A binary indicator variable (lonely) was also generated, where values >6 and <9 were coded as lonely (1), and values ≤6 as not lonely (0) [33]. Respondents missing any of the three items were excluded from the scale.

#### 2.3.5. Perceived Mental Health Need

Perceived mental health need was assessed using a self-reported item that asked participants to indicate their agreement with the statement: “In the past 12 months, I needed help for emotional or mental health problems or challenges such as feeling sad, blue, anxious, or nervous.” Responses were recorded on a six-point Likert scale ranging from 1 (Strongly agree) to 6 (Strongly disagree). To create a binary indicator variable, responses were dichotomized: participants selecting “Strongly agree,” “Agree,” or “Somewhat agree” (values 1–3) were coded as 1, indicating perceived need, while those selecting “Somewhat disagree,” “Disagree,” or “Strongly disagree” (values 4–6) were coded as 0. Missing responses were preserved during processing and subsequently excluded from regression analyses to maintain analytic integrity.

#### 2.3.6. Insurance

Health insurance status was derived from a multi-response item asking participants to indicate the source(s) of their current health insurance coverage. Respondents could select multiple options, including coverage through parents, employers, student plans, government programs, embassies, or self-purchased plans. Two additional response options captured lack of coverage, “I do not have any health insurance coverage,” and uncertainty, “I am uncertain about whether I have health insurance”.

Responses were recoded into a single indicator to create a binary insurance status variable for analysis. Participants were classified as insured (coded as 1) if they selected any coverage source (options 2–8 or 10). Those who indicated no insurance (option 1) or uncertainty about coverage (option 9) were classified as uninsured (coded as 0). The missing cases were retained as a separate category to preserve sample size; however, this group was not included in the substantive interpretation of findings.

#### 2.3.7. Treatment Efficacy (Medication and Therapy)

Beliefs about mental health treatment efficacy were assessed using two items: one regarding medication and one regarding counseling or therapy. Respondents were asked: (1) “How helpful on average do you think medication would be for you if you were having mental or emotional health problems?” and (2) “How helpful on average do you think therapy or counseling would be for you if you were having mental or emotional health problems?” Response options for both items included the following: “Very helpful,” “Helpful,” “Somewhat helpful,” and “Not helpful.” For each, a binary variable was created where responses of “Very helpful,” “Helpful,” or “Somewhat helpful” were coded as 1 (indicating a belief that the treatment would be helpful), and “Not helpful” was coded as 0. The missing cases were retained as a separate category to preserve sample size; however, this group was not included in the substantive interpretation of findings. This approach allowed for the inclusion of treatment belief indicators in multivariable analyses without compromising the integrity of results due to listwise deletion.

#### 2.3.8. Awareness of Campus Mental Health Services

Awareness of available mental health services on campus was assessed using the following item: “If I needed to seek professional help for my mental or emotional health, I would know where to go on my campus.” Responses were collected using a six-point Likert scale, ranging from 1 = Strongly agree to 6 = Strongly disagree. In the 2020–2021 and 2021–2022 survey waves, this item was pre-coded into a binary format. To ensure consistency across years, a harmonized binary variable was used in which students who expressed any level of agreement (Strongly agree, Agree, or Somewhat agree) were coded as 1 “aware”, and those who expressed any level of disagreement “Somewhat disagree, Disagree, or Strongly disagree” were coded as 0 “not aware”.

Missing responses were recoded as a distinct category. These cases were retained for descriptive analysis but omitted from inferential models and interpretation to maintain analytical precision while preserving overall sample size.

#### 2.3.9. Perceived Social Support

Perceived social support was assessed using the item: “My social relationships are supportive and rewarding,” rated on a 7-point Likert scale ranging from 1 (Strongly disagree) to 7 (Strongly agree). To facilitate analysis, responses were recoded into a binary variable. Responses of 5 (Slightly agree), 6 (Agree), or 7 (Strongly agree) were coded as 1, indicating perceived social support. Responses from 1 to 4 (ranging from Strongly disagree to Neutral) were coded as 0, indicating low or uncertain support. Missing responses were retained as a distinct category for descriptive purposes but were excluded from inferential analyses.

#### 2.3.10. Sociodemographic Covariates

Participants self-reported demographic characteristics, including age (categorized as 18–22, 23–25, 26–30, or 31+ years), sex/gender identity (male, female, or other), and degree level (undergraduate, graduate, or non-degree). Race/ethnicity was restricted to students who identified as Arab American or Arab/Middle Eastern. These variables were included as covariates in multivariable models to adjust for demographic variation in mental health outcomes and digital service utilization. Participants responded to the question “Are you an international student?” with binary response options (1 = Yes, 0 = No). This variable was used to distinguish Arab American students from Arab/Middle Eastern international students in all analyses. Missing responses were excluded from the final analytic sample.

### 2.4. Statistical Analysis

All analyses were conducted using Stata version 18.5. Descriptive analyses were conducted to compare the prevalence of depression, anxiety, and loneliness between Arab American and AME international college students during the post-COVID-19 recovery period (academic years 2022–2023 and 2023–2024). Logistic regression analyses were conducted to examine the bidirectional associations between loneliness and both depression and anxiety, adjusting for a comprehensive set of covariates. The assumptions of the logistic regression analyses (including linearity of the logit, multicollinearity, and influential outliers were tested) and no violations were detected. To examine the relationship between loneliness, perceived need, and the use of DMH services, weighted logistic regression analysis was used. To further contextualize these relationships within a comprehensive framework, we conducted Generalized Structural Equation Modeling (GSEM) to test the full conceptual model (Figure 2). Model fit was evaluated using McFadden’s pseudo R^2^, derived from the log pseudolikelihood values, consistent with prior applications of GSEM in clinical research [34].

## 3. Results

### 3.1. Sample Characteristics

The study sample included 3249 Arab/Middle Eastern students enrolled at U.S. colleges and universities. Of these, 81.9% identified as Arab American (*n* = 2662) and 18.1% as Arab/Middle Eastern international students (*n* = 587). Table 1 presents the demographic and enabling characteristics of these two groups. Gender and academic level distributions differed between groups. Arab American students were predominantly female, younger (67% aged 18–22), and mostly undergrads (74%). In contrast, AME international students were more evenly distributed across age groups, included a higher proportion of males (48%), and were primarily graduate students (59%).

With respect to enabling factors, approximately 71% of Arab Americans and 69% of international students reported having insurance coverage. Awareness of campus services was reported by 59% and 57%, respectively, while perceptions of social support were somewhat higher among Arab Americans (75%) than international students (65%). Beliefs about treatment efficacy were broadly similar across groups, with a considerable proportion of both groups indicating “unknown”.

Comparative analyses of included vs. excluded students were also conducted during revision (Appendix A Table A1), showing systematic differences in missingness that may affect generalizability.

### 3.2. Prevalence Comparisons by Group

The analyses in this section address Hypothesis 1, which compared Arab American and AME international students. Hypotheses 2 and 3 were examined using the full Arab/Middle Eastern sample without separating by group.

#### 3.2.1. Depression, Anxiety, and Loneliness

Descriptive analyses were conducted to compare the prevalence of depression, anxiety, and loneliness between Arab American and AME international college students during the post-COVID-19 recovery period (academic years 2022–2023 and 2023–2024). As shown in Table 1, 44.33% of Arab American students reported symptoms of depression, compared to 40.89% of AME international students, indicating a slightly higher prevalence among Arab Americans. Similarly, anxiety symptoms were more common among Arab American students (40.01%) than AME international students (36.97%). In contrast, feelings of loneliness were nearly identical between the two groups, with 56.20% of Arab American and 56.90% of AME international students reporting loneliness.

#### 3.2.2. Correlates of Depression and Anxiety

As shown in Table 2, loneliness was significantly associated with increased odds of both depression and anxiety symptoms. In the fully adjusted model, students who reported loneliness had 2.19 times higher odds of depression (OR = 2.19, 95% CI: 1.66–2.88, *p* < 0.001) and 1.99 times higher odds of anxiety (OR = 1.99, 95% CI: 1.49–2.65, *p* < 0.001) compared to those who did not report loneliness.

Conversely, symptoms of depression were also associated with higher odds of reporting loneliness (OR = 2.16, 95% CI: 1.65–2.84, *p* < 0.001), as were symptoms of anxiety (OR = 1.97, 95% CI: 1.49–2.60, *p* < 0.001), supporting the hypothesized bidirectional relationship. In addition, the models demonstrated strong comorbidity between depression and anxiety: students with anxiety had 12.00 times greater odds of reporting depression (OR = 12.00, 95% CI: 9.06–15.91, *p* < 0.001), while those with depression had 12.07 times greater odds of reporting anxiety (OR = 12.07, 95% CI: 9.12–15.97, *p* < 0.001).

#### 3.2.3. Loneliness, Perceived Need, and Digital Mental Health Utilization

**Figure 3:**
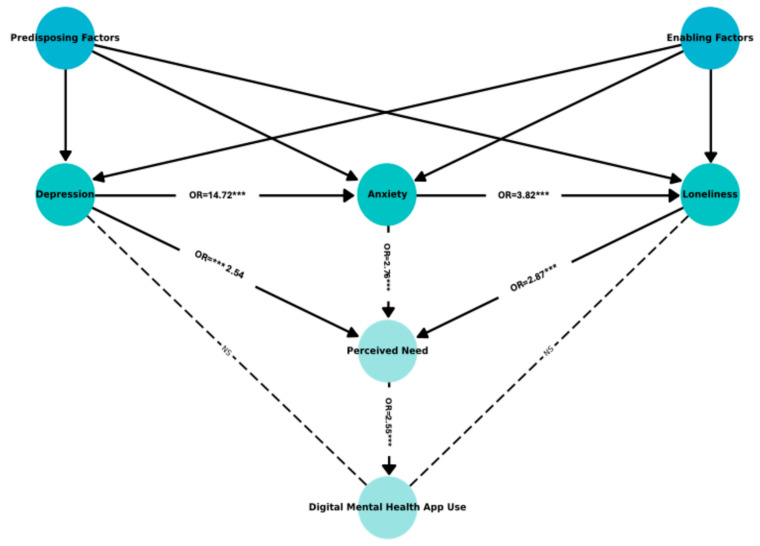
Estimated model with significant relationships (*** *p* < 0.01; NS = not significant).

The path analysis results (Figure 3 and Appendix A Table A2) confirmed the conceptual structure in Figure 2 i.e., depression (β = 0.93, OR = 2.54, *p* < 0.001), anxiety (β = 1.02, OR = 2.77, *p* < 0.001), and loneliness (β = 1.05, OR = 2.86, *p* < 0.001) were all significant predictors of perceived need. Perceived need significantly predicted DMH use (β = 0.94, OR = 2.56, *p* < 0.001). However, the direct path from loneliness to DMH use was not statistically significant (β = 0.12, OR = 1.13, *p* = 0.685). An interaction term testing the moderating effect of loneliness on the relationship between perceived need and DMH use (loneliness × perceived need) was also non-significant (β = −0.29, OR = 0.75, *p* = 0.381), suggesting that loneliness does not significantly moderate this association.

## 4. Discussion

The hypothesis that AME international students would report higher rates of mental health concerns was not supported. Instead, Arab American students reported slightly higher levels of depression and anxiety, while loneliness was similar between the two groups. These modest differences may reflect the unique sociocultural and structural stressors that Arab American students face, such as racial discrimination, acculturative pressures, and systemic marginalization within U.S. educational settings [35,36]. In fact, these results challenge the common assumption that international students are always more vulnerable to psychological distress simply because they are adapting to a new culture and living away from family support.

Several factors may explain these findings. Arab American students often experience ongoing minority stress, including racism, Islamophobia, and cultural marginalization, which can negatively impact mental health [37,38]. Unlike international students, who may see their time in the U.S. as temporary, Arab Americans often navigate these systemic challenges as part of their long-term daily lives. Unlike international students, who may see their time in the U.S. as temporary, Arab Americans often navigate these systemic challenges as part of their long-term daily lives. These cultural stressors were not directly measured in this study, but prior research suggests they are likely important contributors to Arab American students’ elevated rates of depression and anxiety. Future research should incorporate direct measures of minority stress and acculturative pressures to better explain these group differences. It’s also possible that AME international students represent a self-selected group with stronger coping skills, higher resilience, and greater motivation qualities that may help them manage emotional distress more effectively. Additionally, they may view their challenges as part of a short-term academic journey, which could reduce the overall emotional burden [39].

Differences in how students disclose their symptoms could also play a role. Arab American students may feel more comfortable acknowledging and reporting psychological distress, while international students might be more likely to downplay or withhold these experiences because of cultural norms or lower mental health literacy. In addition, international students often have access to campus resources and support services specifically designed for them, such as international student advisors and culturally tailored programs. These supports may help reduce some of the psychological challenges they face during their studies. Overall, these findings suggest that simply being an international student or a U.S. citizen does not determine vulnerability to mental health challenges. Instead, factors such as minority stress, discrimination, and access to culturally responsive support services may play a much larger role in shaping mental health outcomes for Arab-identifying students in the U.S.

To further explore group-level differences, we conducted multivariable regression analyses comparing depression and anxiety outcomes among AME international students and Arab American students. After adjusting for sociodemographic characteristics, beliefs about treatment effectiveness, and factors related to access, we found no statistically significant differences between the two groups. This suggests that the initial differences in prevalence might be explained more by underlying structural or individual factors rather than ethnicity or international status alone. Similar within-heritage differences have also been observed in other student populations. Asian American students, for example, have been shown to face greater exposure to model minority stereotypes and more adverse mental health effects compared to their international peers, highlighting how citizenship and sociocultural positioning can influence psychological outcomes even within the same ethnic group [40].

However, the findings from the present study support Hypothesis 2, indicating a reciprocal relationship between loneliness and mental health conditions like depression and anxiety. Loneliness can both contribute to emotional distress and emerge as a response to it, creating a reinforcing cycle. This bidirectional connection is consistent with earlier research showing that loneliness can act both as a cause and a consequence of psychological distress [41,42], and it has also been observed in other student populations; for example, passively sensed data from U.S. college students found that loneliness was strongly associated with subsequent depressive symptoms, while sleep and physical activity served as protective factors [43]. Although our findings align with this perspective, the cross-sectional design of this study limits causal interpretation. Longitudinal research is needed to clarify the temporal sequence and directionality of these associations. While our models accounted for many sociodemographic and perception-related factors, these results should still be interpreted with caution. Other unmeasured influences, such as financial strain, chronic health problems, or stress related to immigration, may also play an important role in shaping mental health outcomes for this population [35,36,44].

The SEM results showed no significant direct link between symptoms of depression and anxiety and the use of DMH services. Similarly, the direct path from loneliness to DMH use was not significant, suggesting that loneliness influences service utilization only indirectly through perceived need. This suggests that simply having symptoms isn’t enough to motivate students to seek help; what matters more is whether they recognize and acknowledge a need for care. This mediating role of perceived need aligns with previous research highlighting its crucial role in mental health service use [26,45,46]. Evidence from non-U.S. samples reinforces this pattern; for instance, Chinese residents during COVID-19 who reported higher loneliness also reported greater perceived mental health need and higher service use [47]. Similarly, research on international students in the U.S. during the pandemic found that experiences of discrimination were closely tied to loneliness, anxiety, and depressive symptoms, underscoring the vulnerabilities of culturally marginalized groups [8].

Importantly, the gap we observed between experiencing symptoms and actually using DMH services points to ongoing access barriers, which may be especially challenging for AME students. Cultural stigma, limited awareness of available services, and skepticism about the usefulness of digital tools have all been identified as common obstacles for these populations [22,48]. Our findings also align with prior research showing that engagement with digital mental health (DMH) interventions is shaped by a range of psychosocial and perceptual factors. For example, Borghouts et al. (2021) [26] identified stigma, low digital literacy, and doubts about usefulness as common barriers to engagement across DMH platforms. These themes were reflected in our data, particularly among AME students who reported limited perceived need and low uptake of DMH services despite experiencing significant psychological distress [26]. These findings highlight the importance of culturally responsive strategies that not only reduce stigma but also help students see digital mental health services as relevant, trustworthy, and accessible.

## 5. Limitations

This study has several limitations that should be acknowledged. First, because the study is based on cross-sectional data, we cannot make causal inferences about the relationships between variables. While we observed associations between loneliness, depression, and anxiety, these findings do not establish the temporal direction of effects. Longitudinal studies are needed to confirm the sequence and potential causality of these relationships.

Second, although the Healthy Minds Study provides a large and diverse dataset, this analysis was limited to data from two academic years (2022–2023 and 2023–2024). This is because key measures, including loneliness and DMH service use, were part of optional modules that institutions could choose whether to include. As a result, not all institutions administered these questions, and students at those schools were excluded from the analyses. This may have introduced selection bias, as our final sample may overrepresent students from institutions that opted to collect more detailed mental health data. Additionally, students who were excluded due to missing responses, particularly on treatment belief items, may differ systematically from those who completed the survey, potentially skewing our understanding of help-seeking attitudes in the broader AME student population. Our analysis also did not account for institutional variation in the availability and promotion of digital mental health services, which may affect utilization patterns and further limit the generalizability of these findings. Consequently, our findings might not fully reflect all students across participating institutions. Future research should encourage more consistent use of these modules to improve generalizability.

Although non-response weights were applied, the exclusion of institutions that did not administer optional modules may still bias findings. Comparative analyses of included vs. excluded students (Appendix A, Table A1) indicated systematic differences in missingness (e.g., on treatment beliefs and insurance coverage), which may introduce selection bias. This suggests that findings may not fully generalize to all Arab/Middle Eastern students across institutions. Future research should incorporate post-stratification or multiple imputation as formal sensitivity analyses to further test robustness.

Third, while survey weights were used to improve representativeness, the relatively small number of unweighted AME international students might have limited our ability to detect group-level differences.

Fourth, all data were self-reported, which raises the potential for recall errors or social desirability bias. Although the broader dataset includes other relevant factors, such as stigma, financial stress, prior mental health diagnoses, and details about campus-specific support systems, these variables were not included in this analysis. Their exclusion may have limited the range of explanatory variables and introduced residual confounding.

Finally, some constructs were assessed using single-item measures, such as perceived need for care and perceived social support, which may not fully capture the complexity of students’ attitudes, experiences, or motivations related to mental health help-seeking. Future studies should consider using validated multidimensional instruments and incorporating a broader set of psychosocial predictors to more fully capture the factors shaping mental health and help-seeking among AME students.

## 6. Implications

A central finding from this study is that students’ perceived need for support plays a more crucial role in their use of DMH services than simply experiencing symptoms. This underscores the importance of increasing mental health awareness, reducing stigma, and making campus resources more visible and accessible. Helping students recognize when they need support is a critical first step toward engaging with mental health services.

The findings from this study offer several meaningful insights for mental health service planning and campus policies aimed at supporting AME students in U.S. higher education. Importantly, the results suggest that citizenship status alone does not define vulnerability to mental health challenges. Instead, tailored interventions should address the unique psychosocial stressors experienced by both AME international and Arab American students. The bidirectional relationship between loneliness and internalizing symptoms like depression and anxiety highlights the need for preventive strategies. Fostering more socially connected and inclusive campus environments can help reduce feelings of isolation, ease psychological distress, and ultimately promote better mental health outcomes.

To be truly effective, mental health services must move beyond a one-size-fits-all approach. Colleges and universities should implement strategies at multiple levels: individual, institutional, and cultural. This might include developing culturally tailored digital tools, incorporating routine mental health screenings into student health services, and training staff to deliver care that is culturally responsive and inclusive. Key factors such as personalization, ease of navigation, and human support have been shown to enhance engagement with digital mental health interventions [26]. These insights underscore the need for user-centered and culturally responsive design when scaling up DMH services for AME student populations.

Moreover, universities need to address broader structural issues affecting AME students, such as anti-Arab racism, Islamophobia, and systemic marginalization. These factors can harm students’ well-being and create significant barriers to care. Building trust through culturally humble and respectful service delivery is crucial to making mental health support not only accessible but also effective and equitable for AME student populations [49,50,51].

Future research should adopt longitudinal designs to clarify causal and bidirectional relationships between loneliness, mental health symptoms, perceived need, and DMH service use. Incorporating qualitative or mixed-methods approaches could also help uncover cultural attitudes, stigma, and contextual barriers that shape help-seeking behaviors. Studies should further explore campus-level and structural factors, including culturally responsive services and institutional support, and examine less-studied areas like acculturation stress. To improve generalizability and equity, national datasets should ensure adequate representation of AME students and consistently include relevant variables across survey waves.

## 7. Conclusions

This study provides timely insight into the mental health experiences of Arab American and AME international college students during the post-COVID-19 recovery period. Contrary to common assumptions, Arab American students reported slightly higher levels of depression and anxiety than their international peers, although feelings of loneliness were similar across both groups. Multivariable analyses revealed a strong two-way relationship between loneliness and mental health symptoms, highlighting the critical role of social isolation in shaping mental health outcomes.

One of the most important findings was the role of perceived need, which emerged as a key factor connecting mental health symptoms to DMH service use. This highlights the importance of mental health literacy and students’ ability to recognize their own distress when deciding to seek support. While loneliness itself was not directly linked to DMH service use, its strong connection to depression, anxiety, and perceived need suggests that targeting social connection may indirectly increase engagement with services.

Overall, these findings underscore the importance of developing culturally responsive, campus-based mental health strategies that strengthen social support and enhance access to digital resources. Addressing the unique psychosocial challenges faced by Arab-identifying students, particularly through inclusive policies and tailored programs, may help reduce disparities in mental health care and promote more equitable outcomes across diverse student populations.

## Figures and Tables

**Table 1:** Prevalence of Mental Health Indicators and Demographic Characteristics Among Arab American and AME International Students (2022–2024).

Variable	Arab American (*n* = 2662)	AME International (*n* = 587)
Age		
Age: 18–22 Years	1794 (67.39%)	179 (30.49%)
Age: 23–25 Years	357 (13.41%)	100 (17.04%)
Age: 26–30 Years	295 (11.08%)	151 (25.72%)
Age: 31+ Years	216 (8.11%)	157 (26.75%)
Unknown	0 (0.0%)	0 (0.0%)
Gender		
Male	697 (26.18%)	282 (48.04%)
Female	1789 (67.21%)	293 (49.91%)
Other	155 (5.82%)	11 (1.87%)
Unknown	21 (0.79%)	1 (0.17%)
Degree Level		
Undergraduate	1980 (74.38%)	228 (38.84%)
Graduate	560 (21.04%)	345 (58.77%)
Non-degree	34 (1.28%)	6 (1.02%)
Unknown	88 (3.31%)	8 (1.36%)
Beliefs about Treatment Efficacy		
Beliefs about Medication		
Helpful	898 (33.73%)	215 (36.63%)
Not helpful	367 (13.79%)	58 (9.88%)
Unknown	1397 (52.48%)	314 (53.49%)
Beliefs about Therapy		
Helpful	1177 (44.21%)	256 (43.61%)
Not helpful	89 (3.34%)	18 (3.07%)
Unknown	1396 (52.44%)	313 (53.32%)
Insurance		
Has insurance	1899 (71.34%)	404 (68.82%)
No insurance	101 (3.79%)	16 (2.73%)
Unknown	662 (24.87%)	167 (28.45%)
Awareness of Campus Services		
Aware	1566 (58.83%)	335 (57.07%)
Lack of awareness	570 (21.41%)	124 (21.12%)
Unknown	526 (19.76%)	128 (21.81%)
Perceived Social Support		
Supportive	1986 (74.61%)	381 (64.91%)
Non-supportive	671 (25.21%)	205 (34.92%)
Unknown	5 (0.19%)	1 (0.17%)
Mental Health Indicators		
Depression		
Yes	1180 (44.33%)	240 (40.89%)
No	1482 (55.67%)	347 (59.11%)
Anxiety		
Yes	1065 (40.01%)	217 (36.97%)
No	1597 (59.99%)	370 (63.03%)
Loneliness		
Lonely (UCLA score: 6–9)	1496 (56.2%)	334 (56.9%)
Not Lonely (UCLA score: 3–5)	1166 (43.8%)	253 (43.1%)
Perceived need		
Need	1761 (66.15%)	430 (73.25%)
No need	901 (33.85%)	157 (26.75%)
Digital Mental Health Service Use		
Yes	260 (9.77%)	64 (10.9%)
No	2402 (90.23%)	523 (89.1%)

**Table 2:** Multivariable Logistic Regression Predicting Depression and Anxiety (*n* = 3249).

Predictor	Depression		Anxiety	
	OR [95% CI]	*p*-Value	OR [95% CI]	*p*-Value
Mental Health Indicators				
Loneliness	2.19 [1.66, 2.88]	***	1.99 [1.49, 2.65]	***
Anxiety	12.00 [9.06, 15.91]	***	—	—
Depression	—	—	12.07 [9.12, 15.97]	***
Age-group (ref: 18 to 22 years)				
23–25 Years	1.05 [0.69, 1.59]	NS	0.95 [0.63, 1.44]	NS
26–30 Years	0.83 [0.46, 1.51]	NS	0.72 [0.44, 1.17]	NS
31+ Years	0.90 [0.54, 1.50]	NS	0.51 [0.28, 0.92]	**
Gender (ref: Male)				
Female	1.36 [1.02, 1.81]	**	1.47 [1.11, 1.96]	***
Other	2.40 [1.47, 3.93]	***	2.34 [1.32, 4.15]	***
(Unknown) †	3.09 [1.22, 7.86]	**	1.27 [0.32, 5.04]	NS
Race (ref: Arab American)				
AME International	1.09 [0.75, 1.58]	NS	1.01 [0.70, 1.44]	NS
Degree Level (ref: Graduate)				
Undergraduate Student	1.36 [0.90, 2.06]	NS	0.84 [0.57, 1.23]	NS
Non-Degree	1.10 [0.40, 2.99]	NS	0.27 [0.06, 1.11]	*
Unknown † (degree)	1.63 [0.80, 3.35]	NS	0.70 [0.26, 1.90]	NS
Beliefs about Treatment Efficacy				
Medication Helpfulness (ref: Not helpful)				
Helpful	1.31 [0.79, 2.18]	NS	1.56 [0.99, 2.46]	*
Therapy Helpfulness (ref: Not helpful)				
Helpful	0.46 [0.19, 1.09]	*	0.83 [0.37, 1.88]	NS
Insurance (ref: No Insurance)				
Has Insurance	0.62 [0.30, 1.28]	NS	0.77 [0.36, 1.63]	NS
(Unknown) †	0.85 [0.40, 1.80]	NS	0.67 [0.31, 1.44]	NS
Aware of Campus Services (ref: Not aware)				
Aware	0.75 [0.54, 1.04]	*	0.50 [0.35, 0.70]	***
(Unknown) †	0.76 [0.48, 1.20]	NS	0.47 [0.28, 0.77]	***
Perceived social support (ref: Not supportive)				
Supportive	0.59 [0.43, 0.81]	***	0.87 [0.64, 1.20]	NS
(Unknown) †	0.17 [0.03, 0.94]	**	0.25 [0.04, 1.50]	NS
Perceived need (ref: No)				
Need	2.06 [1.53, 2.79]	***	2.68 [1.97, 3.66]	***
Bidirectional Associations: Loneliness as an Outcome				
Depression → Loneliness	2.16 [1.65, 2.84]	***	—	—
Anxiety → Loneliness	—	—	1.97 [1.49, 2.60]	***

† Indicates preserved missing value category. * *p* < 0.10, ** *p* < 0.05, *** *p* < 0.01 indicate levels of statistical significance. NS = Not Statistically Significant.

## Data Availability

Data is available upon reasonable request to authors pending approval from HMS.

## Appendix A

**Table A1:** Descriptive Characteristics of Included and Excluded Arab/Middle Eastern (AME) Student Respondents.

		Included Sample *n* = 3249		Excluded Sample *n* = 586	
Variable	Category	Frequency	Percent (%)	Frequency	Percent (%)
Utilized Digital Mental Health Service					
	Yes	324	9.97		
	No	2925	90.03		
Depression Status					
	Yes	1420	43.71		
	No	1829	56.29		
Anxiety Status					
	Yes	1282	39.45		
	No	1967	60.55		
Loneliness					
	Yes	1830	56.31		
	No	1419	43.69		
Perceived Need					
	Yes	2191	67.42		
	No	1058	32.58		
Race					
	Arab American	2662	81.93	441	75.26
	AME International	587	18.07	124	21.16
	Unknown	0	0.00	21	3.58
Age Group					
	18–22 Years	1973	60.73	383	65.36
	23–25 Years	457	14.07	58	9.9
	26–30 Years	446	13.73	69	11.77
	31+ Years	373	11.48	76	12.97
Gender					
	Male	979	30.14	204	34.81
	Female	2082	64.08	346	59.04
	Other	166	5.11	26	4.44
	Unknown	22	0.68	10	1.71
Degree Level					
	Undergraduate	2208	67.96	322	54.95
	Graduate	905	27.85	118	20.14
	Non-degree	40	1.23	16	2.73
	Unknown	96	2.95	130	22.18
Medication Helps					
	Helpful	1113	34.26	16	2.73
	Not helpful	425	13.08	5	0.85
	Unknown	1711	52.66	565	96.42
Therapy Helps					
	Helpful	1435	44.15	18	3.07
	Not helpful	107	3.29	2	0.34
	Unknown	1707	52.56	566	96.59
Insurance Coverage					
	Yes	2303	70.89	40	6.83
	No	117	3.6	2	0.34
	Unknown	829	25.52	544	92.83
Awareness of Services					
	Aware	694	21.36	525	89.59
	Not Aware	1901	58.52	20	3.41
	Unknown	654	20.13	41	7
Perceived Social Support					
	Supportive	2367	72.88	294	50.17
	Non-supportive	876	26.96	100	17.06
	Unknown	6	0.18	192	32.76

**Table A2:** Summary of Significant Predictors from SEM Analysis.

Outcome	Predictor	Direction	Significance	Significance
Depression Status	Female gender (ref: Male)	Positive	*p* < 0.001	***
	Other gender identity (ref: Male)	Positive	*p* < 0.001	***
	Age 23–25 (ref: 18–22Y)	Positive	*p* = 0.092	*
	Age 31+ (ref: 18–22Y)	Negative	*p* = 0.006	**
	Undergraduate (ref: Graduate)	Positive	*p* = 0.085	*
	Awareness of services (ref: No)	Negative	*p* < 0.001	***
	Perceived social support: Yes (ref: Not Supportive)	Negative	*p* < 0.001	***
Anxiety Status	Depression Status	Positive	*p* < 0.001	***
	Female gender (ref: Male)	Positive	*p* < 0.001	***
	Other gender identity (ref: Male)	Positive	*p* < 0.001	***
	Age 31+ (ref: 18–22Y)	Negative	*p* = 0.018	**
	Non-degree (ref: Graduate)	Negative	*p* < 0.001	***
	Awareness of services (ref: No)	Negative	*p* < 0.001	***
	Perceived social support: Yes (ref: Not Supportive)	Negative	*p* < 0.001	***
Loneliness	Anxiety Status	Positive	*p* < 0.001	***
	Other gender identity (ref: Male)	Positive	*p* = 0.001	**
	Age 23–25 (ref: 18–22Y)	Negative	*p* = 0.084	*
	Age 31+ (ref: 18–22Y)	Negative	*p* = 0.000	***
	Perceived social support: Yes (ref: Not Supportive)	Negative	*p* < 0.001	***
Perceived Need	Depression Status	Positive	*p* < 0.001	***
	Anxiety Status	Positive	*p* < 0.001	***
	Loneliness	Positive	*p* < 0.001	***
	Female gender (ref: Male)	Positive	*p* = 0.000	***
	Other gender identity (ref: Male)	Positive	*p* = 0.000	***
	International student status: Yes	Positive	*p* < 0.001	***
	Undergraduate (ref: Graduate)	Negative	*p* = 0.001	***
	Therapy is helpful (ref: Not helpful	Positive	*p* = 0.001	***
	Awareness of services (ref: No)	Positive	*p* = 0.041	**
Digital MH Use	Anxiety Status	Positive	*p* = 0.069	*
	Perceived Need	Positive	*p* = 0.000	***
	Female gender (ref: Male)	Positive	*p* = 0.000	***
	Other gender identity (ref: Male)	Positive	*p* = 0.045	**
	Age 23–25 (ref: 18–22Y)	Positive	*p* = 0.066	*
	Age 31+ (ref: 18–22Y)	Positive	*p* = 0.067	*

* *p* < 0.10, ** *p* < 0.05, *** *p* < 0.01 indicate levels of statistical significance.
